# Prolonged grief disorder in ICD-11 and *DSM*-5-TR: Challenges and controversies

**DOI:** 10.1177/00048674231154206

**Published:** 2023-02-06

**Authors:** Maarten C Eisma

**Affiliations:** Department of Clinical Psychology and Experimental Psychopathology, University of Groningen, Groningen, the Netherlands

**Keywords:** Complicated grief, persistent complex bereavement disorder, medicalization, pharmacotherapy, stigma

## Abstract

Prolonged grief disorder has recently been added to the *International Classification of Diseases*, 11th edition and the *Diagnostic and Statistical Manual of Mental Disorders* 5, Text Revision. This historical development is often presented as a linear process culminating in the inclusion of valid, clinically relevant prolonged grief disorder criteria in diagnostic handbooks. The present contribution provides an overview of work contradicting this dominant narrative. First, I show that the developmental history of prolonged grief disorder has been nonlinear and that this yields questions on generalizability and problems with measurement of the newest criteria sets. Second, I highlight an important gap in the validity evidence: the distinction of prolonged grief disorder from normal grief. Third, I discuss concerns relating to the societal effects of the inclusion of prolonged grief disorder in diagnostic handbooks, including the medicalization of grief, development and adverse effects of pharmacotherapy and stigmatization. A more realistic, balanced view on the history, validity and societal impact of prolonged grief disorder appears appropriate. I recommend stringent validation of assessment instruments for prolonged grief disorder, convergence of criteria-sets, closing gaps in validity evidence and developing strategies to mitigate the negative effects of grief diagnoses.

## Introduction

Grief experts generally agree that a substantial minority of bereaved people experience severe, persistent and disabling grief, which requires indicated treatment (e.g. Boelen et al., 2020; Prigerson et al., 2021b; Simon et al., 2020). Over the years, multiple proposals have been drafted of a disorder characterized by such grief responses, termed complicated grief disorder (Horowitz et al., 1997), prolonged grief disorder (PGD; e.g. Prigerson et al., 2009), complicated grief (e.g. Shear et al., 2011) and persistent complex bereavement disorder (American Psychiatric Association, 2013). Recently, two different diagnoses termed PGD have been formally included in the *International Classification of Diseases*, 11th edition (*ICD*-11: World Health Organization, 2018) and the *Diagnostic and Statistical Manual of Mental Disorders* 5, Text Revision (*DSM*-5-TR: American Psychiatric Association, 2022: Table 1).

**Table 1.** Diagnostic criteria for PGD per *ICD*-11 and *DSM*-5-TR.

ICD-11 PGD criteria		*DSM*-5-TR PGD criteria	
A. Event criterion	History of bereavement following the death of a partner, parent, child or other person close to the bereaved.	A. Event and time criteria	The death, at least 12 months ago, of a person who was close to the bereaved (for children and adolescents, at least 6 months ago).
B. Separation distress	A persistent and pervasive grief response characterized by one of the following symptoms: 1. Longing for the deceased 2. Persistent preoccupation of the deceased	B. Separation distress	Since the death, the development of a persistent grief response characterized by one or both of the following symptoms, which have been present most days to a clinically significant degree. In addition, the symptom(s) have occurred nearly every day for at least the last month: 1. Intense yearning/longing for the deceased person. 2. Preoccupation with thoughts or memories of the deceased person (in children and adolescents, preoccupation may focus on the circumstances of the death)
C. Intense emotional pain	Accompanied by intense emotional pain, for example, sadness, guilt, anger, denial and blame Difficulty accepting the death Feeling that one has lost a part of one’s self An inability to experience positive mood Emotional numbness Difficulty engaging with social or other activities	C. Cognitive, emotional and behavioral symptoms	Since the death, at least three of the following symptoms have been present most days to a clinically significant degree. In addition, the symptoms have occurred nearly every day for at least the last month: 1. Identity disruption (e.g. feeling that a part of oneself has died) since the death 2. Marked sense of disbelief about the death 3. Avoidance of reminders that the person is dead (in children and adolescents, may be characterized by efforts to avoid reminders) 4. Intense emotional pain (e.g. anger, bitterness, sorrow) related to the death 5. Difficulty reintegrating into one’s relationships and activities after the death (e.g. problems engaging with friends, pursuing interests or planning for the future) 6. Emotional numbness (absence or marked reduction of emotional experience) as a result of the death 7. Feeling that life is meaningless as a result of the death 8. Intense loneliness as a result of the death
D. Functional impairment criterion	The disturbance results in significant impairment in personal, family, social, educational, occupational or other important areas of functioning. If functioning is maintained, it is only through significant additional effort.	D. Functional impairment criterion	The disturbance causes clinically significant distress or impairment in social, occupational or other important areas of functioning.
E. Cultural and time criteria	The pervasive grief response has persisted for an atypically long period of time following the loss, markedly exceeding expected social, cultural or religious norms for the individual’s culture and context. Grief responses lasting for less than 6 months, and for longer periods in some cultural contexts, should not be regarded as meeting this requirement.	E. Cultural criterion	The duration and severity of the bereavement reaction clearly exceeds expected social, cultural or religious norms for the individual’s culture and context.
		F. Relation to other mental disorders	The symptoms are not better explained by major depressive disorder, posttraumatic stress disorder or another mental disorder, or attributable to the physiological effects of a substance (e.g. medication, alcohol) or another medical condition.

*Note.ICD*-11: the *International Classification of Diseases*, 11th edition; *DSM*-5-TR: the *Diagnostic and Statistical Manual of Mental Disorders* 5, Text Revision; PGD: prolonged grief disorder.

Proponents have argued the necessity of establishing a PGD diagnosis. However, potential disadvantages, validity evidence gaps and controversial issues relating to the inclusion of PGD in diagnostic handbooks are generally given short shrift. Awareness of such issues is critically important to improve the implementation and development of PGD diagnoses and assess and mitigate potential adverse societal effects. Therefore, following a brief discussion of support for PGD’s inclusion in diagnostic handbooks, I will provide an overview of key challenges and controversies related to this development.

## Support for PGD in ICD-11 and *DSM*-5-TR

Advocates of the inclusion of PGD in diagnostic handbooks often interpret research on prolonged grief symptoms (using various scales and conceptualizations) as evidence in support of the validity of PGD (e.g. Boelen et al., 2020; Killikelly and Maercker, 2017; Prigerson et al., 2021b; Simon et al., 2020). Historically, such research is often focused on construct validity (e.g. dimensionality of prolonged grief symptoms; e.g. O’Connor et al., 2010; Simon et al., 2011), convergent validity (correlations of prolonged grief symptoms with related disorders; e.g. Aoyama et al., 2018; Simon et al., 2007), divergent validity (distinctiveness of prolonged grief symptoms from symptoms of related disorders; e.g. Boelen & van den Bout, 2005; Dillen et al., 2009) and criterion validity (predictive value of prolonged grief symptoms for other relevant constructs: e.g. Boelen and Prigerson, 2007; Prigerson et al., 2009). Moreover, the clinical relevance of PGD is often illustrated by other means, such as the results from a randomized controlled trial (RCT) demonstrating that grief-specific therapy is more effective in treating prolonged grief symptoms than a depression-focused therapy (Shear et al., 2014). Recent studies have replicated some (but by no means all) of these findings using measures of (approximations of) symptoms of PGD per *ICD*-11 and *DSM*-5-TR (e.g. Boelen and Lenferink, 2020, 2022; Haneveld et al., 2022; Lenferink et al., 2022; Prigerson et al., 2021a).

In addition, advocates have argued that the inclusion of PGD in diagnostic handbooks will have positive consequences for researchers, clinicians and bereaved persons (e.g. Boelen et al., 2020). For example, it could provide an impetus to investigate risk and protective factors, maintaining mechanisms and care for severe grief reactions. It will also foster the identification of, communication about and the provision and reimbursement of targeted, more effective care for bereaved people needing help following loss.

## PGD in *ICD*-11 and *DSM*-5-TR: challenges and controversies

Despite substantial validity evidence and compelling arguments in favor of the inclusion of PGD in diagnostic handbooks, this development has not gone uncontested (e.g. Bandini, 2015; Cacciatore and Francis, 2022; Eisma et al., 2020; Stroebe et al., 2000; Wakefield, 2012). Below, three key points of contention are discussed (a) the non-linear history of PGD (and problems of generalizability and measurement), (b) the unclear distinction of PGD from normal grief and (c) potential negative societal consequences.

### Non-linear history of PGD

The first challenge to the support for PGD originates from its developmental history. An assumption underlying most aforementioned empirical evidence is that research on prior proposals of pathological grief directly informs the validity of current PGD criteria sets. However, the history of PGD is non-linear: past proposals did not systematically build on each other to logically culminate in current diagnoses. Instead, previous proposals show substantial differences in symptom count and content, time (since loss) criteria and diagnostic algorithms compared to current PGD criteria sets (Eisma et al., 2020, 2022; Lenferink et al., 2021; Stelzer et al., 2020b). Consequently, frequently used measures for prolonged grief symptoms, such as the Inventory of Complicated Grief (ICG) and the Prolonged Grief Scale 13 (PG-13), do not comprehensively assess PGD per *ICD*-11 or *DSM*-5-TR (Lenferink et al., 2022; O’Connor et al., 2020; Treml et al., 2020). This leads to uncertainty whether past research on the characteristics of pathological grief generalize to current criteria sets. For example, PGD per *ICD*-11 diverges from prior proposals of pathological grief, affecting important phenomenological characteristics such as diagnostic agreement (Eisma et al., 2020). Moreover, despite some pioneering empirical comparisons (e.g. Haneveld et al., 2022; Lenferink et al., 2022), it remains relatively unclear whether findings on characteristics of the two current PGD criteria sets generalize to each other.

Concerns about generalizability are compounded in a cross-cultural context. Most evidence on the validity of past and current grief disorder conceptualizations is derived from samples from Western countries, and, to a lesser extent, from East Asia. Whether PGD per *ICD*-11 and *DSM*-5-TR are valid diagnoses in other parts of the world is therefore an open question. There are indications that there is substantial heterogeneity in the experience, interpretation and reporting of prolonged grief symptoms across cultures. For example, a recent review showed that Asian bereaved adults generally report higher prolonged grief symptom levels than European and American bereaved adults (Stelzer et al., 2020b). However, Balinese adults bereaved due to traffic accidents reported remarkably low prolonged grief symptom levels (Djelantik et al., 2021). The authors argued that Balinese cultural rituals and notions reduced the likelihood of endorsing strong negative emotions, preventing the development of prolonged grief symptoms. Other populations may be at heightened risk for PGD, but cultural characteristics may make the application of instruments assessing this condition challenging. For example, the indigenous people of Australia and New Zealand are at risk for severe grief reactions, due to high rates of all-cause mortality, suicidal deaths and exposure to other traumatic life-events (Spiwak et al., 2012). Yet, the holistic view of health used by these cultural groups may increase the likelihood that ‘standard’ instruments do not adequately assess indications of PGD (cf. Le Grande et al., 2017). To surmise, it cannot be assumed that diagnostic criteria developed for North American and European populations will be valid and accurate in different cultural contexts.

Therefore, comprehensive, international systematic research on these new criteria sets is critical to gather accurate information about the characteristics of PGD per *ICD*-11 and *DSM*-5-TR. A challenge to doing so, is that both criteria sets were new when introduced, implying that there were no validated instruments available to assess these criteria. A first solution to this problem is to use approximations of criteria sets (e.g. Boelen and Lenferink, 2020; Comtesse et al., 2020; Mauro et al., 2019, 2022; Prigerson et al., 2021a). That is, items from past measures of pathological grief and other disorders are selected to approximate new diagnostic criteria. If carefully applied, this method can shed light on the extent to which characteristics of previous criteria sets generalize to new criteria sets (Eisma et al., 2020). For example, Haneveld et al. (2022) demonstrated that the estimated rate of diagnostic agreement between PGD per *ICD*-11 and PGD per *DSM*-5-TR substantially increased when aligning the time criteria for both diagnoses to 1 year post-loss. However, a drawback of approximating symptoms is the degree of uncertainty regarding the extent to which chosen items accurately reflect recent criteria. For optimal results, transparent reporting of how selected items map on current symptoms and acknowledgment of the limitations of this approach, is critical.

A second solution to this problem is the development and validation of new instruments to assess the latest conceptualizations of PGD. However, developing such instruments and applying them in research has proven challenging. For example, many symptoms of PGD *ICD*-11 do not allow for unambiguous interpretations (Eisma et al., 2020). Researchers can easily interpret one word-criteria such as ‘blame’, ‘anger’ and ‘denial’ differently, which may in turn affect results on the characteristics of PGD. For example, self-blame is much more prevalent following loss than blaming others (Davis et al., 1995) and repetitive thought on self-blame relate to increases of prolonged grief symptoms, whereas repetitive thought on blaming others does not (Eisma et al., 2022). Relatedly, there is no diagnostic algorithm for PGD per *ICD*-11, making it difficult to determine the optimal number of accessory symptoms for research purposes (Eisma et al., 2020; Stelzer et al., 2020b).

Despite these difficulties, one validated scale exists to screen for PGD per *ICD*-11, the International Prolonged Grief Disorder Scale (IPGDS: Killikelly et al., 2020). Interestingly, there have been multiple studies on the cross-cultural validity of the IPGDS, including the exploration of the value of developing additional items to capture culture-specific grief experiences (e.g. Killikelly et al., 2020, 2021; Stelzer et al., 2020a). Another instrument screens for both PGD per *ICD*-11 and *DSM*-5-TR: the Traumatic Grief Inventory Self Report Plus (TGI-SR +: Lenferink et al., 2022), facilitating research comparing both criteria sets. The newly devised Prolonged Grief Scale 13 Revised (PG-13-R) may also prove useful to measure symptoms of PGD per *DSM*-5-TR (Prigerson et al., 2021a), but still awaits formal psychometric evaluation. Validated structured clinical interviews, critical to establish PGD diagnoses (Horowitz, 2006), are not yet publicly available.

Using new measures, we can investigate whether and under what circumstances key characteristics of past and current grief disorders are similar or different, to address concerns about generalizability of findings across criteria sets. Moreover, by examining how different interpretations of PGD *ICD*-11 criteria and algorithms affect its phenomenology and comparability to other grief disorders, we can collect data that may improve consensus for future criteria sets (Eisma et al., 2020).

### Unclear distinction of PGD from normal grief

A second, more fundamental challenge to the support for PGD is that a key question on the divergent validity of PGD is unanswered: How can we distinguish PGD from normal grief? (Stroebe et al., 2000; Wakefield, 2012). The distinction of pathological grief from normal grief is fundamental to the recognition of PGD as diagnosis, as per definition a disorder should reflect a dysfunction of the individual, rather than form a natural response to the external environment (Wakefield, 2012). According to *DSM*-5-TR (American Psychiatric Association, 2022), a mental disorder is *‘a syndrome characterized by clinically significant disturbance in an individual’s cognition, emotion regulation, or behavior that reflects a dysfunction in the psychological, biological, or development processes underlying mental functioning*’. Following current conceptualizations of PGD (Table 1), it may be distinguished from normal grief by qualitatively different subjective experiences, a greater intensity, longer duration and resulting functional impairment.

The evidence that PGD is characterized by qualitatively different experiences than normal grief is yet unconvincing. In one relevant study by Boelen and van den Bout (2008), a selection of prolonged grief symptoms assessed with the ICR–Revised (ICG-R, Prigerson and Jacobs, 2001) was shown to load on a different factor than items from the Texas Revised Inventory of Grief (TRIG, Faschingbauer et al., 1987). This was interpreted as evidence that pathological grief can be discerned from normal grief. However, the TRIG was originally developed to assess both normative and pathological grief responses, obscuring the meaning of these findings. Two other, similar studies applying taxometric analyses to items of versions of the ICG-R supported a dimensional rather than a categorical conceptualization of grief (Holland et al., 2008; Kliem et al., 2018). This suggests that pathological grief is not characterized by the presence or absence of specific symptoms, but instead differs from normal grief only in severity. The problem of discerning grief from pathological grief is compounded by the fact that we have no consensus-based definition of normal grief (Stroebe et al., 2000) and that there is substantial variability in content of scales proposed to assess normal grief. When we do not agree on what normal grief is, how can we distinguish it from PGD?

Distinguishing pathological grief from normal grief in terms of duration and severity may provide a better alternative. For example, based on a normal distribution of data on grief symptoms, one may classify anyone scoring two standard deviations above the mean as experiencing probable PGD. The development of specific cut-off points (e.g. a score above 25 on the ICG: Prigerson et al., 1995) is another example of this approach. One could also use consensus-based cut-offs, of which the 6- and 12-month time-criteria of PGD are well-known examples. Nevertheless, considering that both grief severity and duration are continuous variables, any criteria derived in this manner will be arbitrary (cf. Wakefield, 2012). Being on the far end of a continuum of grief severity or duration does not automatically imply that the grief you experience is pathological.

Another method to discern normal grief from PGD is through the presence or absence of functional impairment. For example, according to *DSM*-5-TR (American Psychiatric Association, 2022), *‘the (grief) disturbance must cause clinically significant distress or impairment in social, occupational, or other important areas of functioning’.* While this may be a clinically useful approach, it is partially subject to the same arbitrariness as the severity and duration distinction. For example, distress is likely to follow a normal distribution; what level of distress would be considered clinically significant?

Addressing the unclear distinction between normal grief and PGD poses a major challenge to the validity of PGD. Expert consensus on what normal grief is and how normal grief experiences are different from PGD symptoms may lay the groundwork for a clearer empirical differentiation of both phenomena. In the related field of health psychology, researchers have made major advancements in defining a range of psychological constructs through an iterative, consensus-based approach (Michie et al., 2013). However, it should be noted that a difference between normal variations of human experiences and mental disorder remains unclear for many related stress-related and affective disorders despite attempts to improve definitions of disorders (Wakefield, 2016). Improving conceptual clarity may thus not provide a completely satisfactory solution to this fundamental problem of diagnostic classification.

### Potential negative societal consequences

The third challenge to PGD originates from a variety of expected negative societal consequences. Three main concerns are the medicalization of normal grief, risks of novel pharmacotherapies and stigmatization of people diagnosed with PGD.

#### Medicalization of normal grief

First, many researchers have voiced concerns that introduction of PGD in diagnostic handbooks will lead to medicalization of normal grief responses and overdiagnosis (e.g. Bandini, 2015; Cacciatore and Francis, 2022; Wakefield, 2012). Such concerns often revolve around the observation that for some groups of bereaved persons, severe and persistent grief may be a natural response and therefore not represent a medical disorder. Often, such concerns are dismissed by observations that the prevalence of PGD is relatively low in general population samples, which reduces odds of overdiagnosis (Boelen et al., 2020; Prigerson et al., 2021b; Prigerson and Maciejewski, 2022). Nevertheless, for some groups of bereaved adults, severe and persistent grief appears a normal (i.e. frequently occurring) response to an abnormal life-event. According to a recent meta-analysis, the estimated prevalence of pathological grief is very high in people who experience unnatural deaths (49%: Djelantik et al., 2020). Similarly, the loss of a child or parent relates to more severe and persistent grief responses (for a review: Burke and Neimeyer, 2013). For example, approximately one-in-three parents who lost a child due to cancer experienced probable PGD in a national Swedish sample (Pohlkamp et al., 2019). Therefore, diagnosing these people with PGD would imply labeling normal grief variations as a medical disorder.

Notably, most grief experts with concerns about medicalization would likely agree that appropriate care should be provided to distressed bereaved people. However, they believe that labeling people with a medical disorder is not an appropriate means of achieving this goal. Nevertheless, in many countries, a medical label is required to receive reimbursed mental health care. It appears justified to disagree with diagnosing people showing normal responses to an extraordinary negative life-event. However, the implication, withholding reimbursed effective care from severely distressed bereaved persons because their grief can in principle not be labeled a disorder, is also difficult to defend. Therefore, unless we drastically change how health care is organized, researchers and clinicians have to choose between two ‘evils’ when making a choice on diagnosing PGD in some bereaved persons.

#### Risks of pharmacotherapy

Second, some researchers are concerned about the impetus that the establishment of PGD will provide to the development and application of medication for bereaved persons. While antidepressants are often prescribed for bereaved adults (Lacasse and Cacciatore, 2014), results on their effectiveness have been equivocal (Bui et al., 2012). A rare, large placebo-controlled RCT demonstrated no efficacy of citalopram as a treatment for prolonged grief symptoms (Shear et al., 2016). However, it suggests that adding citalopram to an effective cognitive behavioral therapy (CBT) treatment may alleviate co-occurring depressive symptoms. Another ongoing placebo-controlled RCT will examine the effects of naltrexone on PGD (Gang et al., 2021). Naltrexone is prescribed based on the idea that PGD resembles addiction wherein the bereaved person continues to seek a connection with the deceased. Naltrexone may disrupt this behavior, reducing core symptoms of PGD, such as yearning. However, naltrexone will likely also reduce feelings of social connectedness with close living others (Thieleman et al., 2022). Thereby, it could interfere with the provision of social support, often regarded as critical to psychological adaptation to loss. Evidently, the limited empirical support for the benefits of medication for bereaved adults and potential negative side effects warrant great caution from researchers and clinicians when prescribing and testing medication for people diagnosed with this condition.

#### Stigmatization

Third, laypeople, researchers and clinicians have voiced concerns that the establishment of PGD will lead to stigmatization (Breen et al., 2015; Dietl et al., 2018; Ogden and Simmonds, 2014). Stigma is defined as the co-occurrence of labeling, stereotyping, separation, status loss and discrimination in a context in which power is exercised (Link and Phelan, 2001). Stigmatization can have major negative consequences, including increases in depression and suicidality (Carpiniello and Pinna, 2017), reduced help seeking (Clement et al., 2015) and premature termination of mental health treatments (Sirey et al., 2001).

A series of vignette-based experimental studies conducted in the Netherlands, Germany and Australia has demonstrated that the general public stigmatizes people with PGD symptoms and diagnoses (vs people with non-clinical grief) more (Dennis et al., 2022; Eisma, 2018; Eisma et al., 2019; Gonschor et al., 2020). That is, people with PGD are attributed more negative traits and elicit more negative emotions and a greater desire for social distance. Results align with a survey demonstrating that people with severe grief report more negative social reactions (Johnson et al., 2009).

While stigma is generally acknowledged as a negative outcome of the establishment of PGD, most proponents of this development regard it as a necessary evil (e.g. Boelen et al., 2020; Prigerson et al., 2021b; Simon et al., 2020). Nevertheless, the manifold potential negative effects of stigmatization warrant a systematic and concerted effort to better understand and counter the stigma associated with PGD. Providing effective treatment for PGD will likely reduce the associated stigma (Gonschor et al., 2020). Media campaigns and direct contact with individuals with a psychiatric diagnosis may also help disconfirm mental health stereotypes and reduce associated feelings and behaviors (Morgan et al., 2018).

## Conclusion

The inclusion of PGD in the *ICD*-11 and *DSM*-5-TR has introduced consensus-based definitions of pathological grief. While there are both empirical and practical arguments in support of these developments, it has introduced new challenges and has not solved existing challenges and controversies. A first major challenge to the inclusion of PGD in diagnostic handbooks is a lack of consistency in past and current definitions of pathological grief. This necessitates the development and psychometric evaluation of new measures and systematic international research using such measures to establish the validity and cross-cultural applicability of PGD. A second major challenge is the unclear distinction of PGD from normal grief. While difficult to address, one critical step in this process may be to develop a consensus-based definition of normal grief. A third major challenge consists of potential negative societal consequences, including medicalization, application of possibly harmful pharmacotherapies, and stigmatization. Some of these societal effects may be mitigated, for example by expressing caution in prescribing medication to bereaved adults or by reducing stigma toward PGD through psychotherapy and public education. Continued awareness of challenges and controversies relating to the inclusion of PGD in diagnostic handbooks is critical to improve the implementation and development of these new diagnoses.
